# Bronchopneumonia in wild boar *(Sus scrofa)* caused by *Rhodococcus equi* carrying the VapB type 8 plasmid

**DOI:** 10.1186/1756-0500-6-111

**Published:** 2013-03-25

**Authors:** Agueda Castagna de Vargas, Fernanda Monego, Letícia Trevisan Gressler, Sônia de Avila Botton, Andrea Maria Lazzari, Mateus Matiuzzi da Costa, Roselene Ecco, Márcio Garcia Ribeiro, Gustavo Henrique Batista Lara, Shinji Takai

**Affiliations:** 1 Department of Preventive Veterinary Medicine, Universidade Federal de Santa Maria, UFSM, 97105-900, Santa Maria, State of Rio Grande do Sul, Brazil; 2 Department of Microbiology, Universidade do Contestado- UnC, Santa Catarina, Brazil; 3 Department of Veterinary Medicine, União Pioneira de Integração Social – UPIS, Brasília, Brazil; 4 Department of Veterinary Medicine, Universidade Federal do Vale do São Francisco - UNIVASF, Petrolina, State of Pernambuco, Brazil; 5 Department of Veterinary Clinic and Cirugic, School of Veterinary, Universidade Federal de Minas Gerais -UFMG, State of Minas Gerais, Brazil; 6 Department of Veterinary Hygiene and Public Health, School of Veterinary Medicine and Animal Sciences, Universidade Estadual Paulista - UNESP, Box 560, Botucatu, State of Sao Paulo, Code 18618-970, Brazil; 7 Department of Animal Hygiene, School of Veterinary Medicine and Animal Sciences, Kitasato University, Kitasato, Japan

**Keywords:** *Rhodococcus equi*, Wild boar (*Sus scrofa*), VapB plasmid, Bronchopneumonia

## Abstract

**Background:**

*Rhodococcus equi* is associated with pyogranulomatous infections, especially in foals, and this bacterium has also emerged as a pathogen for humans, particularly immunocompromised patients. *R. equi* infections in pigs, wild boar (*Sus scrofa*) and humans are mainly due to strains carrying the intermediate virulence (VapB) plasmid. In Brazil, *R. equi* carrying the VapB type 8 plasmid is the most common type recovered from humans co-infected with the human immunodeficiency virus (HIV). *R. equi* infection in pigs and wild boar is restricted predominantly to the lymphatic system, without any reports of pulmonary manifestations.

**Findings:**

This report describes the microbiological and histopathological findings, and molecular characterization of *R. equi* in two bronchopneumonia cases in wild boar using PCR and plasmid profile analysis by digestion with restriction endonucleases. The histological findings were suggestive of pyogranulomatous infection, and the plasmid profile of both *R. equi* isolates enabled the characterization of the strains as VapB type 8.

**Conclusions:**

This is the first report of bronchopneumonia in wild boar due to *R. equi*. The detection of the VapB type 8 plasmid in *R. equi* isolates emphasize that wild boar may be a potential source of pathogenic *R. equi* strains for humans.

## Findings

### Background

*Rhodococcus equi* is a well-recognized gram-positive facultative intracellular pathogen. It primarily infects domestic animals, particularly foals. *R. equi* has also emerged as an opportunistic pathogen of humans, particularly immunocompromised patients [1,2]. The bacterium has been isolated from the feces and intestines of various herbivorous and omnivorous species, including cattle, sheep, horses, deer, goats, and pigs [3,4]. The most common manifestation of rhodococcosis in humans and animals is a progressive pyogranulomatous bronchopneumonia, characterized by purulent abscesses and cavitation [3].

The virulence of *R. equi* is attributed to several factors, including a capsule, cholesterol oxidase and, in particular, plasmid-encoded virulence-associated proteins (Vaps) [1]. These plasmids determine the pathogenicity of *R. equi,* as they are associated with the survival of the bacterium inside macrophages [3]. Three levels of virulence of *R. equi* are recognized: virulent (VapA), intermediately virulent (VapB) and avirulent. Virulent *R. equi* strains are predominantly found in horses, whereas intermediate virulence isolates have been more often observed in pigs [5], HIV-positive human patients [6], and, recently, in wild boar with and without lymphadenitis [7,8]. The avirulent *R. equi* strains do not express VapA or VapB [9]. The VapB plasmids contains *vap*B and other *vap* genes (*vap*J, *vap*K1, *vap*K2, and *vap*M) [10,11].

In Brazil, the first wild boar (*Sus scrofa*) bred for commercial purposes were introduced into the state of Rio Grande do Sul from Europe approximately in 1980 [12]. They were subsequently introduced into other Brazilian states as the commercialization of wild boar meat production increased. This report describes the first case of bronchopneumonia in wild boar caused by *R. equi* carrying a VapB type 8 plasmid.

### Materials and methods

#### Ethical statement

The present study was approved by Ethical Committee of Animals (number 192/09-CEUA), of School of Veterinary Medicine and Animal Science – UNESP, Botucatu, SP, Brazil.

#### Animals

Two 70 to 80-day-old wild boar from a breeding farm in Distrito Federal (DF), Brazil, showed clinical signs of pulmonary infection. The animals had exhibited delayed physical developmental, decreased appetite, lethargy, persistent coughing, difficulty breathing, body temperature approximately of 40°C and râles of moderate intensity on thoracic auscultation. Samples of the lungs and pulmonary lymph nodes were collected at necropsy and subjected to microbiological and histopathological analyses.

#### Diagnosis methods

For histopathology, the specimens were fixed in 10% neutral buffered formalin and, embedded in paraffin, and the sections were stained with hematoxylin and eosin (HE). The lung samples of both animals were cultured, and bacterial identification was based on the observed morphological, staining and biochemical characteristics [13]. The results were confirmed using an *R. equi*-specific polymerase chain reaction (PCR), as previously described [14]. Bacterial DNA was obtained from a pure colony suspended in 100 μL of Milli-Q water, boiled for 7 minutes and centrifuged at 60,000 × g for 4 minutes [15]. A second PCR was used to identify the virulence-associated genes of the isolates, as described previously [16]. Primer 1 (5′-ACAAGACGGTTTCTAAGGCG-3′) and primer 2 (5′-TTGTGCCAGCTACCAGAGCC-3′) were used to detect the virulent (*vapA* gene) strains by amplifying a 550-bp product. Primer 3 (5′-GAATTCGAAAGCGCAAAGGT-3′) and primer 4 (5′-TTCCGTGAACATCGTACTGC -3′) were used to amplify a 650-bp product from intermediately virulent (*vapB* gene) isolates. The virulent strain ATCC 33701p and a human isolate previously characterized as *vap*B-positive were used as the positive controls in all the PCR reactions.

The plasmid types were determined by digestion with restriction endonucleases. *R. equi* plasmid DNA was isolated using a modified alkaline lysis method [17], as described previously [18]. The Plasmid DNA was digested with the restriction endonucleases *EcoRI*, *EcoT22I* and *HindIII*[19]. The fragments were fractionated on 1.0% agarose gels, stained with ethidium bromide and examined under ultraviolet light.

### Results

#### Pathology

**Figure 1:**
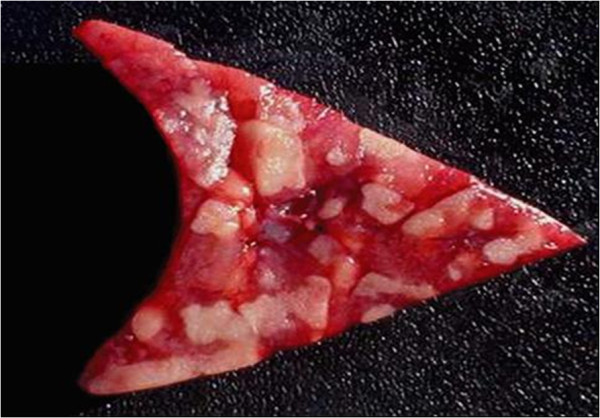
**Pneumonia in lung lobe of wild boar caused by *R. equi *characterized by necrotic multifocal to coalescent lesions, and dark-red consolidated areas.**

**Figure 2:**
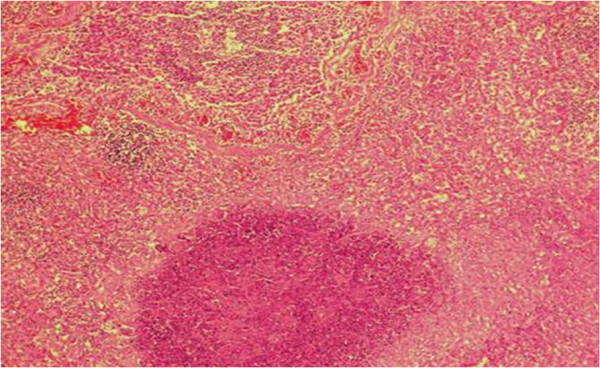
**Necrotic bronchopneumonia caused by *R. equi *in the cranial lung lobe from a wild boar*.*** Note area of necrosis containing micro-organisms surrounded by degenerate neutrophils, epithelioid cells and macrophages (Hematoxylin and eosin, 100X).

At necropsy, the lungs were found to have multifocal to coalescent areas and granulomatous lesions, with dark-red consolidated areas in the cranial lobes and the ventral portion of the caudal lobes (Figure 1), and mucupurulent exudates in the bronchial lumina. The histological examinations revealed marked neutrophil infiltration into the bronchial and bronchiolar lumina. Large areas of necrosis, with bacterial colonies surrounded by degenerate neutrophils, epithelioid macrophages were also observed in the cranial lung lobes (Figure 2). Numerous foamy macrophages and neutrophils within the alveoli and chronic marked bronchopneumonia were also observed. The histological changes observed in the lungs and pulmonary lymph nodes were suggestive of *R. equi* infection [20].

#### Bacterial isolation and molecular characterization

*R. equi* was isolated from the lungs of both boar, and no other bacteria were isolated. The *R. equi* isolates from both wild boar were *vap*B *R. equi*.

#### Plasmid characterization

The plasmid profile of both *R. equi* isolates were those of VapB type 8.

### Discussion

*R. equi* infections in wild boar cause lesions that are similar to those observed in rhodococcosis in pigs, and are generally restricted to the lymphatic tissues, involving the cervical and submaxillary lymph nodes, and the tonsils [11,21]. *R. equi* infection in pigs is associated with immunosuppression and co-infections with other pathogens [4], although *R. equi* have been isolated from swine with and without lymphadenitis [8,22]. In the cases describe herein, *R. equi* was identified as a primary cause of pulmonary lesions in the wild boar. Intermediately virulent *R. equi* strains are commonly found in submandibular lymph nodes in pigs, immunosuppressed humans [5], and more recently, wild boar [7,8]. In Hungary, *R. equi* was isolated from 14% and 12.4% of the submaxillary lymph nodes of pigs and wild boar, respectively [7,21], and these studies found the *vapB* gene in 26.8% of the *R. equi* isolates from pigs [21] and 25.6% from wild boar [7]. In Japan, vapB-positive *R. equi* were isolated with high prevalence from 368 (93.9%) lymph nodes from pigs [23]. Another study in Japan, described recently isolation of 45 (52%) *R. equi* strains from submaxillary lymph nodes of 86 wild boar. From these strains were found vapB-positive in 21 (24.0%) strains, predominantly types 2, 1 and 4 plasmids, while *vapA* gene was found in 1 (1.0%) strain, and 23 (27.0%) remaining isolates were avirulent [24]. Currently, a Brazilian study of the virulence genes and plasmid profiles of the *R. equi* isolates from the lymph nodes of slaughtered wild boar found that 63.2% of the isolates contained the *vapB* gene, and the majority was identified as carrying type 8 plasmids [8]. Interestingly, this same plasmid profile (VapB type 8) is the one most frequently found in HIV-positive patients in Brazil [25], suggesting the zoonotic potential of *R. equi* isolates from wild boar. However, the human with rhodococcosis generally have no history of contact with pigs, wild boar or the environments of these species [25]. It is possible that the consumption of undercooked pig and wild boar products may be a route of infection with *R. equi* in humans in some countries [6,8,24,25].

### Conclusions

This is the first report of *R. equi* causing bronchopneumonia in wild boar. Although the role of domestic animals and wildlife in the transmission of *R. equi* to humans remains unclear, the detection of *R. equi* carrying a VapB type 8 plasmid in wild boar emphasizes the possibility that this species may be a potential source of virulent *R. equi* in humans, due to this plasmid profile has been identified in *R. equi* isolates from humans.

### Availability of supporting data

The data set supporting this short report are contain in paper.

## Abbreviations

bp: Base pairs; DNA: Deoxyribonucleic acid; HE: Hematoxylin eosin; HIV: Human immunodeficiency virus; PCR: Polymerase chain reaction; Vap: Virulence-associated proteins

## Competing interests

The authors declare that there are no conflicts of interest in this work.

## Authors’ contributions

This case report was written by ACV, FM, LTG and SAB. It was reviewed by all the authors, particularly MGR and GHBL. Microbiological culture and phenotypic identification were performed by CCK, MMC and AML. Clinical and histopathological examination of animals were performed by RE. Determination of the plasmid types of the *R. equi* isolates was carried out by ST, FM and MGR. All authors read and approved the final version of the manuscript.
